# Physical activity perceptions, barriers, and needs among nursing home residents: an explanatory sequential mixed-methods study

**DOI:** 10.3389/fpubh.2026.1823151

**Published:** 2026-09-03

**Authors:** Jung-A. Kim, Haeyeon Kim, Hye-Young Jang

**Affiliations:** College of Nursing, Hanyang University, Seoul, Republic of Korea

**Keywords:** barriers, mixed-methods study, needs, nursing home residents, older adults, perceptions, physical activity

## Abstract

**Background/objectives:**

Physical activity is essential for maintaining health and preventing functional decline among older adults residing in nursing homes. However, engagement in physical activity remains suboptimal in this population due to multifactorial influences. This study aimed to examine the perceptions, barriers, and needs related to physical activity among nursing home residents.

**Methods:**

An explanatory sequential mixed-methods design was employed from September to December 2024. In the quantitative phase, data were collected from 254 residents across 20 nursing homes in Korea using structured questionnaires assessing physical activity frequency, fall efficacy, outcome expectations, and social support. Hierarchical logistic regression analyses were conducted to identify factors associated with physical activity participation. In the subsequent qualitative phase, in-depth interviews were conducted with 12 residents and analyzed using reflexive thematic analysis. Findings from both phases were integrated to provide a comprehensive interpretation of the contextual influences on physical activity.

**Results:**

Facility size, program frequency, program duration, social support, and education level were significantly associated with physical activity. Residents living in facilities with 30 or more beds were less likely to engage in physical activity. Higher program frequency was positively associated with physical activity participation, whereas program sessions lasting 30 min or longer were negatively associated with participation. Greater perceived social support was also associated with higher physical activity participation. Residents with a middle school education or higher were more likely to engage in physical activity. Qualitative analysis identified five themes: meanings shaped by past experiences; physical activity as restoration; motivating forces amid decline; multilayered barriers; and expectations for improved opportunities. Integration of findings revealed that personal meaning, interpersonal relationships, and institutional contexts dynamically interact to shape physical activity engagement.

**Conclusion:**

Physical activity among nursing home residents is shaped by the dynamic interplay of individual experiences, social support, and environmental conditions. Strategies to promote physical activity should therefore adopt a holistic and context-sensitive approach.

## 1 Introduction

The global population of adults aged 65 years and older is rapidly increasing, leading to a growing number of older adults transitioning to nursing homes due to age-related declines in physical function and independence (1, 2). For nursing home residents, maintaining physical activity is essential for preserving physical function and psychological well-being (3). However, engagement in physical activity commonly declines following institutionalization (4).

Compared with community-dwelling older adults, nursing home residents tend to be older and experience higher levels of dependency due to impairments in activities of daily living and cognitive function (5). Environmental constraints, reduced autonomy, and limited access to structured programs further restrict opportunities for movement, contributing to predominantly sedentary lifestyles (6). The transition to institutional care can also disrupt social relationships and daily routines, increasing risks of depression, loneliness, and psychological distress (7). Regular physical activity is a key strategy for preventing functional decline and maintaining overall health. Evidence indicates that physical activity improves gait ability, reduces fall risk, alleviates depressive and behavioral symptoms, and enhances sleep quality and overall quality of life among nursing home residents (3, 8, 9). Despite these benefits, more than 38–50% of residents report a substantial decrease in activity levels after admission (4). This decline reflects not only individual health conditions, such as chronic disease and fear of falling (6), but also environmental and organizational factors, including insufficient support from caregivers and limited availability of structured exercise programs (10). Although the importance of physical activity is well established, context-specific and sustainable strategies tailored to nursing home environments remain limited (11).

Physical activity engagement in nursing homes is therefore shaped by a complex interplay of individual, environmental, and psychosocial factors. These include measurable determinants, such as health status and facility characteristics, as well as subjective experiences, motivations, and perceived barriers. Studies that rely solely on quantitative indicators provide limited insight into how these factors interact within the lived realities of institutionalized older adults.

To address this gap, the present study employs an integrated mixed methods design. Grounded in a pluralistic perspective that recognizes the coexistence of objective conditions and subjective meanings (12), this approach enables the integration of statistical patterns with residents’ lived experiences. By combining quantitative analysis of associated factors with qualitative exploration of perceptions, motivations, barriers, and expectations, this study aims to generate context-sensitive insights and identify practical strategies to support voluntary and sustained participation in physical activity among nursing home residents. The specific objectives were as follows:

To identify the level of physical activity and associated influencing factors through quantitative analysis.

To explore residents’ experiences of physical activity through qualitative interviews.

To integrate quantitative and qualitative findings to comprehensively analyze residents’ perceptions and needs related to physical activity.

## 2 Materials and methods

### 2.1 Study design

**Figure 1:**
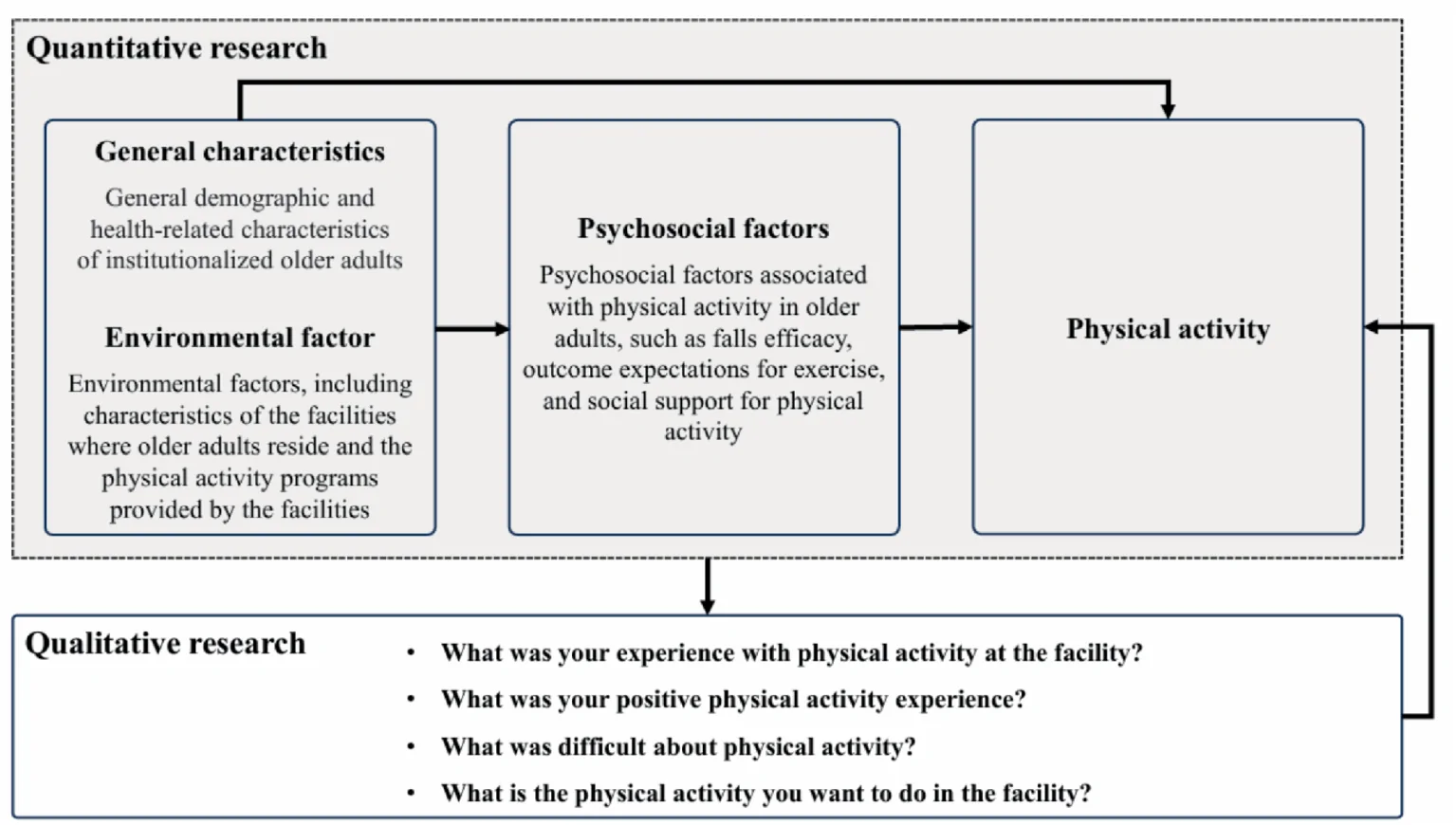
Research design.

This study employed an explanatory sequential mixed-methods design to comprehensively explore nursing home residents’ perceptions and needs regarding physical activity. First, quantitative data were collected to assess levels of physical activity and identify related influencing factors using structured questionnaires. Based on these findings, one-on-one in-depth interviews were conducted to gather qualitative data. Quantitative and qualitative results were then integrated to develop a comprehensive understanding of physical activity among nursing home residents (Figure 1).

### 2.2 Participants

Participants were older adults aged 65 years or older who had resided in a nursing home for at least 1 month, were capable of participating in physical activity, understood the purpose of the study, and voluntarily agreed to participate. The minimum length of stay was determined based on Brooke’s classification of adaptation stages (13), which indicates that confusion during the initial adjustment period to nursing home admission typically occurs within the first few weeks. Individuals who had been diagnosed with dementia at the time of admission according to the primary diagnosis and facility medical records, or who were classified as being at high risk for falls that restricted or contraindicated physical activity participation, were excluded from the study.

The sample size for the quantitative component was calculated using the G*Power 3.1.9.2 program (University of Düsseldorf, Düsseldorf, Germany) for logistic regression analysis with a two-tailed test, an odds ratio (OR) of 0.68 (14), a probability (Pr) (Y = 1|X = 1) under the null hypothesis of 0.50, a significance level of *α* = 0.05, and a statistical power of 0.80. The minimum required sample size was 228. Considering a 10% dropout rate, 254 participants were recruited through convenience sampling. All completed questionnaires (*N* = 254) were included in the final analysis without exclusions.

For the qualitative component, participants were selected using purposive sampling from among those who had completed the quantitative survey and voluntarily agreed to participate in follow-up interviews. In accordance with the principles of adequacy and data saturation in qualitative research, interviews were conducted with a total of 12 residents.

### 2.3 Data collection

Data collection was conducted after obtaining approval from the Institutional Review Board of the researchers’ affiliated institution.

#### 2.3.1 Quantitative data collection

Quantitative data were collected from 254 older adults residing in 20 nursing homes in Korea between September 8 and November 11, 2024. Data collection was conducted by the researchers. After providing a full explanation of the study purpose, significance, and procedures, written informed consent was obtained from participants who voluntarily agreed to participate. A structured questionnaire was then distributed for completion.

Participants were encouraged to complete the questionnaire independently. However, when participants experienced difficulty understanding or responding to specific items, the researcher read the questions aloud and recorded the participants’ responses verbatim. Throughout the data collection process, participants’ visual and auditory conditions were carefully considered. Questionnaires printed in large font were provided as needed, and oral explanations were offered when necessary. For participants using hearing aids, explanations were delivered from a position that facilitated optimal hearing, and comprehension was confirmed prior to proceeding with the survey. Among the survey items, handgrip strength and body mass index (BMI) were measured directly by the researchers.

Handgrip strength was assessed using a hand dynamometer (CAMRY EH-101, China). Measurements were taken twice for each hand while participants maintained a position with the elbow lightly touching the torso at approximately a 90-degree angle. The average value of the two measurements for each hand was used. BMI was calculated by dividing body weight (kg) by the square of height (m^2^). Height was measured in centimeters using a measuring tape while the participant stood upright barefoot or wearing thin socks. Body weight was measured using a digital scale (OMRON HEALTHCARE Co., HBF-222T, China); prior to measurement, the scale was placed on a flat surface and zero-calibrated. Participants stood at the center of the scale, and weight was recorded to the nearest 0.1 kg.

#### 2.3.2 Qualitative data collection

Qualitative data were collected through one-on-one in-depth interviews with 12 residents who had participated in the quantitative survey and voluntarily agreed to the interview after fully understanding its purpose. Interviews were conducted from November 15 to December 10, 2024. Sessions were scheduled based on participants’ convenience and took place in a consultation room within each facility, lasting approximately 1 h.

Prior to each interview, participants were informed of the study purpose, procedures, and audio-recording process, and written consent was obtained. Each interview was conducted by either the first or second author, both of whom were doctoral nursing students. The interviewer took brief field notes and audio-recorded the conversation. Interviews were conducted in a comfortable, open atmosphere to encourage participants to freely share their experiences. Interviews continued until sufficient depth and diversity of experiences were obtained and no substantially new concepts relevant to residents’ perceptions, barriers, and needs regarding physical activity emerged from subsequent interviews.

The interviewers had no prior relationship with the participants and were independent of the nursing homes where the participants resided. They were not involved in the participants’ care or facility management and were therefore not in a position to influence the participants’ care or services. Before each interview, participants were informed that their responses would remain confidential, would be used solely for research purposes, and would not be shared with nursing home staff or administrators. Participants were also informed that there were no right or wrong answers and were encouraged to freely express their experiences and opinions.

### 2.4 Ethical considerations

The study received approval from the Institutional Review Board (IRB No. HYUIRB-202407-022-3). Participants were informed of the study purpose, procedures, voluntary participation, anonymity, confidentiality, and the right to withdraw at any time. All participants received a small gift in appreciation for their participation.

### 2.5 Measures

In the quantitative component, validated instruments were used, and all measures demonstrated acceptable levels of internal consistency. The trustworthiness of the qualitative component was enhanced through the use of a systematically developed interview guide (15).

#### 2.5.1 Quantitative measures

##### 2.5.1.1 General and environmental characteristics

General characteristics included gender, age, education level, length of stay in the facility, number of chronic diseases, visual impairment, hearing impairment, history of falls, pain (current), handgrip strength, and body mass index (BMI). Environmental characteristics of the facility included facility size, staff types, availability of physical activity spaces, presence and types of physical activity equipment, types of personnel responsible for delivering physical activity programs, types of services provided, frequency of physical activity programs per week, and average program duration per session.

##### 2.5.1.2 Physical activity and functional characteristics

Physical activity characteristics were assessed by asking how many times participants engaged in activities such as walking, strolling, or stretching during the past week. Based on previous research identifying walking as the most representative form of physical activity among nursing home residents (16), physical activity frequency was categorized into two groups: ≤3 times per week and ≥4 times per week. This dichotomization was based on evidence that walking four or more times per week for at least 15 min is significantly associated with longevity in older adults (17), supporting the clinical meaningfulness of this threshold.

Functional status was assessed using the Korean Activities of Daily Living scale (K-ADL). The original ADL Index was developed by Katz et al. (18) to assess independence in performing basic activities of daily living. The K-ADL was developed and validated for Korean older adults by Won et al. (19), and its factor structure and measurement invariance were subsequently confirmed by Park and Park (20). The scale consists of 7 items (dressing, washing/grooming, bathing, eating, transferring, toileting, and continence) rated on a 3-point scale (1 = “no help needed” to 3 = “total help needed”). Total scores range from 7 to 21, with higher scores indicating lower levels of daily functioning. Cronbach’s *α* was 0.94 in the original Korean validation (19) and 0.98 in Park and Park (20); in this study, Cronbach’s α was 0.88.

##### 2.5.1.3 Fall efficacy

Fall efficacy was measured using the Korean version of the Fall Efficacy Scale (FES). The original instrument was developed by Tinetti et al. (21) to assess confidence in performing 10 activities of daily living without falling. The Korean version translated and validated by Jang et al. (22) was used in this study. The scale consists of 10 items rated on a 10-point Likert scale (1 = “not confident at all” to 10 = “completely confident”), with higher scores indicating greater confidence in preventing falls and maintaining physical safety. Cronbach’s *α* was 0.90 in both the original (21) and Korean versions (22); in this study, Cronbach’s α was 0.96.

##### 2.5.1.4 Outcome expectations for exercise

Outcome expectations for exercise were assessed using the Korean version of the Outcome Expectations for Exercise Scale-2 (OEE-2). The OEE-2 was developed by Resnick (23) by adding four negative items to the original OEE (24), and the Korean version validated by Choi and Jung (25) was used in this study. The instrument consists of 13 items rated on a 5-point Likert scale, with higher scores indicating greater expectations regarding the benefits of exercise. In the original OEE-2 (23), Cronbach’s *α* was 0.93 for the positive items and 0.80 for the negative items. Choi and Jung (25) reported Cronbach’s α values of 0.73 and 0.63, respectively. In the present study, reliability was 0.93 for the positive items, 0.71 for the negative items, and 0.90 for the total scale.

##### 2.5.1.5 Social support for physical activity

Social support for physical activity was measured using Korean version developed by Roh and Moon (26), based on the original scale developed by Sallis et al. (27). The instrument consists of six items rated on a 5-point Likert scale, with higher scores indicating greater perceived support for physical activity. Cronbach’s *α* was 0.85 in Roh and Moon (26), and 0.94 in the present study.

#### 2.5.2 Qualitative interview guide

Semi-structured interview questions were developed to supplement and further explain the quantitative findings. Key interview questions included: “Tell me about your experiences with physical activity in the facility.” “What benefits have you experienced from participating in physical activity?” “What challenges have you encountered during physical activity?” “What types of physical activities would you like to participate in at the facility?” Additional probing and follow-up questions were used as needed to explore participants’ experiences in greater depth, clarify meanings, and further examine issues that emerged during the interviews.

### 2.6 Data analysis

Quantitative data were analyzed using SPSS/WIN 23.0 (IBM Corp., Armonk, NY, United States). Descriptive statistics (frequencies, percentages, means, standard deviations) were used to summarize general and environmental characteristics as well as psychosocial variables. Differences in physical activity by general and psychosocial characteristics were examined using independent *t*-tests and ANOVA, with Scheffé’s *post-hoc* test, chi-square tests, and Fisher’s exact tests, as appropriate. Hierarchical logistic regression analyses were conducted to identify factors influencing physical activity. Prior to logistic regression analysis, multicollinearity was assessed using tolerance and variance inflation factor (VIF). Tolerance values ranged from 0.33 to 0.87 and VIF values ranged from 1.16 to 3.02, indicating no evidence of problematic multicollinearity.

Qualitative data were transcribed verbatim and analyzed using MAXQDA 24 following the six-phase reflexive thematic analysis approach proposed by Braun and Clarke (28). Each researcher independently read the interview transcripts several times to become familiar with the data and generated initial codes. The research team subsequently held 10 meetings to discuss the rationale for their coding, examine different interpretations, and iteratively refine the codes, subthemes, and themes. Differing interpretations were discussed collaboratively, and analytic decisions were not determined by academic seniority. One doctoral student researcher had prior experience working in a nursing home, which sensitized her to both the importance of physical activity for residents and the practical challenges of its implementation. The team explicitly considered how this experience and their respective perspectives shaped the interpretation of the data.

Mixed-methods integration involved comparing and connecting quantitative and qualitative findings to identify convergences, divergences, and complementary insights.

## 3 Results

### 3.1 Quantitative findings

#### 3.1.1 Participant and environmental characteristics

Among the 254 participants, 67 (26.4%) were men and 187 (73.6%) were women. The mean age was 82.91 ± 7.61 years, and the largest proportion of participants had completed elementary school (31.1%, *n* = 79). The mean length of stay in the facility was 18.39 ± 21.32 months. A total of 198 residents (78.0%) resided in facilities with ≥ 30 beds. All facilities provided designated spaces and equipment for physical activities. The facilities offered an average of 5.08 ± 1.82 physical activity programs per week, and each session lasted an average of 44.39 ± 17.35 min. Participants engaged in physical activity an average of 4.16 ± 2.06 times per week, with 143 (56.3%) engaging in physical activity ≥4 times per week. The mean ADL score was 11.21 ± 3.24 (Table 1).

**Table 1:** Participant and environmental characteristics (*N* = 254).

Characteristics	Categories	*n* (%) or M ± SD
Participants		
Gender	Man	67(26.4)
	Woman	187(73.6)
Age (years)	–	82.91 ± 7.61
Education level	No formal education	67(26.4)
	Elementary school	79(31.1)
	Middle school	49(19.3)
	High school or higher	59(23.2)
Length of stay in the facility (months)	–	18.39 ± 21.32
Chronic diseases (number)	–	3.23 ± 1.48
Visual impairment	Yes	24(9.4)
	No	230(90.6)
Hearing impairment	Yes	24(9.4)
	No	230(90.6)
History of falls	Yes	102(40.2)
	No	152(59.8)
Pain (current)	Yes	83(32.7)
	No	171(67.3)
Handgrip strength (kg)	Right	12.33 ± 11.66
	Left	11.48 ± 7.29
BMI (kg/m^2^)	–	23.41 ± 3.88
ADL	–	11.21 ± 3.24
Physical activity frequency (per week)	–	4.16 ± 2.06
	≤3	111(43.7)
	≥4	143(56.3)
Facility		
Facility size	≤29	56(22.0)
	≥30	198(78.0)
Staff types	Nurse	2.10 ± 2.39
	Nursing assistant	2.54 ± 1.51
	Care worker	29.59 ± 18.60
	Social worker	2.61 ± 1.35
	Physical therapist	0.87 ± 0.84
	Occupational therapist	0.30 ± 0.46
Availability of physical activity spaces	Yes	254(100.0)
	No	0(0.0)
Presence of physical activity equipment	Yes	254(100.0)
	No	0(0.0)
Type of physical activity equipment^†^	Fixed bicycle	204(80.3)
	Running machine	27(10.6)
	Gait training equipment	196(77.2)
	Resistance band	153(60.2)
	Gym ball	135(53.1)
	Others^‡^	47(18.5)
Types of personnel responsible for delivering physical activity programs^†^	Nurse	64(25.2)
	Nursing assistant	46(18.1)
	Care worker	127(50.0)
	Social worker	170(66.9)
	Physical therapist	133(52.4)
	Occupational therapist	76(29.9)
	Guest instructor	78(30.7)
Types of services provided^†^	Gymnastics	251(98.8)
	Walking	251(98.8)
	Ball games	166(65.4)
	Strength training	199(78.3)
	Resistance band exercise	182(71.7)
	Rehabilitation exercise	172(67.7)
	Yoga and stretching	100(39.4)
Frequency of physical activity programs (per week)	–	5.08 ± 1.82
Average program duration per session (minutes)	–	44.39 ± 17.35
	≤29	45(17.7)
	≥30	209(82.3)

Values are presented as number (percentage) or mean ± standard deviation. BMI, body mass index; ADL, activities of daily living. †, Multiple responses; ‡: Body Spider, Standing frame, Badminton racket, Full-body exercise machine, Twist trainer, Upper limb exercise device, Tilt table.

#### 3.1.2 Psychosocial characteristics related to physical activity

The mean fall efficacy score (range: 0–100) was 68.62 ± 21.05. Outcome expectations for exercise (range: 13–65) had a mean score of 48.59 ± 8.55. Perceived social support for physical activity (range: 6–30) had a mean score of 21.38 ± 5.71 (Table 2).

**Table 2:** Descriptive statistics for activities of daily living, fall efficacy, outcome expectations, and social support for physical activity.

Items	Possible range	Actual range	M ± SD
Fall efficacy	10–100	14–100	68.62 ± 21.05
Outcome expectations for exercise	13–65	13–65	48.59 ± 8.55
Social support for physical activity	6–30	6–30	21.38 ± 5.71

Values are presented as raw scores or mean ± standard deviation.

#### 3.1.3 Differences in physical activity by participant characteristics

Physical activity levels significantly differed according to the number of chronic diseases (*t* = −2.125, *p* = 0.035), ADL (*t* = 1.994, *p* = 0.047), social support for physical activity (*t* = −2.814, *p* = 0.005), facility size (χ^2^ = 28.425, *p* < 0.001), and average program duration per session (χ^2^ = 26.938, *p* < 0.001) (Table 3).

**Table 3:** Differences in general and environmental characteristics by frequency of physical activity participation (*N* = 254).

Characteristics	Categories	Frequency of physical activity participation (per week) n (%) or M ± SD		x^2^ or *t*	*p*
		≤3 (*n* = 111)	≥4 (*n* = 143)		
Participants					
Gender	Man	27(24.3)	40(28.0)	0.428^†^	0.567
	Woman	84(75.7)	103(72.0)		
Age (years)		83.11 ± 7.43	82.76 ± 7.76	0.359	0.720
Education level	No formal education	38(34.2)	29(20.3)	7.170	0.067
	Elementary school	34(30.6)	45(31.5)		
	Middle school	18(16.2)	31(21.7)		
	High school or higher	21(18.9)	38(26.6)		
Length of stay in the facility (months)		17.48 ± 18.00	19.10 ± 23.62	−0.600	0.549
Chronic diseases (number)		3.02 ± 1.21	3.40 ± 1.64	−2.125	0.035
Visual impairment	Yes	9(8.1)	15(10.5)	0.414^‡^	0.666
	No	102(91.9)	128(89.5)		
Hearing impairment	Yes	8(7.2)	16(11.2)	1.158^‡^	0.388
	No	103(92.8)	127(88.8)		
History of falls	Yes	43(38.7)	59(41.3)	0.165^‡^	0.701
	No	68(61.3)	84(58.7)		
Pain (current)	Yes	31(27.9)	52(36.4)	2.021^†^	0.178
	No	80(72.1)	91(63.6)		
Handgrip strength (kg)	Left	11.36 ± 6.53	11.56 ± 7.86	−0.215	0.830
	Right	11.83 ± 7.11	12.72 ± 14.23	−0.604	0.547
BMI (kg/m^2^)		23.41 ± 3.19	23.40 ± 4.35	0.012	0.990
ADL		11.67 ± 3.31	10.85 ± 3.16	1.994	0.047
Fall efficacy		67.28 ± 20.66	69.66 ± 21.37	−0.893	0.373
Outcome expectations for exercise		48.38 ± 7.29	48.76 ± 9.43	−0.366	0.723
Social support for physical activity		20.25 ± 5.61	22.26 ± 5.66	−2.814	0.005
Facility					
Facility size	≤29	7(6.3)	49(34.3)	28.425	<0.001
	≥30	104(93.7)	94(65.7)		
Frequency of physical activity programs (per week)		4.88 ± 2.11	5.24 ± 1.55	−1.485	0.070
Average program duration per session (min)	≤29	4(3.6)	41(28.7)	26.938^‡^	<0.001
	≥30	107(96.4)	102(71.3)		

Values are presented as number (percentage) or mean ± standard deviation. BMI, body mass index; ADL, activities of daily living. ^†^, Fisher–Freeman–Halton’s exact test; ^‡^, Fisher’s exact test.

#### 3.1.4 Factors associated with physical activity

In Model 1, which included general characteristics of residents and facility factors, education level, ADL, facility size, frequency of program per week, and average program duration per session were significant predictors. Compared with residents with no formal education, those with middle school and high school or higher had 2.818 times (95% CI: 1.135–6.996) and 3.561 times (95% CI: 1.426–8.896) higher physical activity frequency, respectively. Lower ADL was associated with a decrease in physical activity frequency (OR = 0.896, 95% CI: 0.815–0.985). Residents in facilities with 30 or more beds had lower physical activity frequency (OR = 0.158, 95% CI: 0.050–0.501) than those in facilities with fewer than 29 beds. Higher frequency of programs was associated with increased physical activity (OR = 1.275, 95% CI: 1.067–1.522). When program sessions lasted ≥30 min, the frequency of physical activity decreased by 0.175 times (95% CI: 0.039–0.782).

In Model 2, which added psychosocial variables (social support, outcome expectations, fall efficacy), significant predictors included education level, facility size, frequency of program per week, average program duration per session, and social support. Middle school and high school or higher graduates were 3.194 times (95% CI: 1.228–8.310) and 3.936 times (95% CI: 1.543–10.042) more likely to engage in physical activity than those with no formal education, respectively. Residents in facilities with 30 or more beds were 0.159 times (95% CI: 0.049–0.515) less likely to engage in physical activity. Higher frequency of programs per week increased physical activity frequency by 1.266 times (95% CI: 1.056–1.519). Program duration per session lasting ≥30 min reduced physical activity frequency by 0.183 times (95% CI: 0.040–0.832). Higher perceived social support was associated with a 1.064-fold increase in physical activity frequency (95% CI: 1.006–1.126). The model fit was significant (χ^2^ = 72.081, *p* < 0.001), with an explanatory power of 33.1% and a prediction accuracy of 71.7% (Table 4).

**Table 4:** Multivariable logistic regression analysis of factors associated with physical activity.

Characteristics	Categories	Model 1						Model 2					
		*B*	SE	*p*	OR	95% CI		*B*	SE	*p*	OR	95% CI	
						Lower	Upper					Lower	Upper
(Constant)		3.629	2.740	0.185	35.676			3.687	2.924	0.207	39.930		
Participants													
Gender	Man	Reference	–	–	–	–	–	–	–	–	–	–	–
	Woman	−0.475	0.411	0.247	0.622	0.278	1.391	−0.527	0.424	0.214	0.591	0.257	1.356
Age (years)		0.005	0.021	0.806	1.005	0.964	1.048	0.001	0.022	0.996	1.001	0.959	1.044
Education level	No education	Reference	–	–	–	–	–	–	–	–	–	–	–
	Elementary school	0.548	0.402	0.173	1.729	0.787	3.799	0.552	0.412	0.180	1.737	0.774	3.899
	Middle school	1.036	0.464	0.026	2.818	1.135	6.996	1.161	0.488	0.017	3.194	1.228	8.310
	High school or higher	1.207	0.467	0.007	3.561	1.426	8.896	1.370	0.478	0.004	3.936	1.543	10.042
Length of stay in the facility (months)		−0.007	0.007	0.370	0.993	0.979	1.008	−0.005	0.008	0.484	0.995	0.980	1.010
Chronic diseases (number)		0.134	0.118	0.258	1.143	0.906	1.442	0.135	0.120	0.259	1.144	0.905	1.447
Visual impairment	Yes	Reference	–	–	–	–	–	–	–	–	–	–	–
	No	0.485	0.629	0.440	1.625	0.474	5.570	0.573	0.631	0.363	1.774	0.515	6.110
Hearing impairment	Yes	Reference	–	–	–	–	–	–	–	–	–	–	–
	No	0.125	0.562	0.824	1.133	0.376	3.412	0.060	0.563	0.916	1.061	0.352	3.197
History of falls	Yes	Reference	–	–	–	–	–	–	–	–	–	–	–
	No	0.081	0.335	0.808	1.085	0.562	2.093	0.103	0.343	0.765	1.108	0.566	2.171
Pain (current)	Yes	Reference	–	–	–	–	–						
	No	0.066	0.399	0.869	1.068	0.489	2.333	0.067	0.405	0.868	1.070	0.484	2.365
Handgrip strength (kg)	Left	−0.036	0.034	0.290	0.964	0.902	1.031	−0.034	0.035	0.322	0.966	0.903	1.034
	Right	−0.003	0.019	0.859	0.997	0.961	1.034	−0.006	0.019	0.740	0.994	0.958	1.031
BMI (kg/m^2^)		−0.067	0.043	0.122	0.935	0.859	1.018	−0.072	0.044	0.100	0.931	0.854	1.014
ADL		−0.110	0.048	0.023	0.896	0.815	0.985	−0.100	0.052	0.055	0.905	0.817	1.002
Facility													
Facility size	≤29	Reference	–	–	–	–	–	–	–	–	–	–	–
	≥30	−1.844	0.589	0.002	0.158	0.050	0.501	−1.837	0.599	0.002	0.159	0.049	0.515
Frequency of physical activity programs (per week)		0.243	0.091	0.007	1.275	1.067	1.522	0.236	0.093	0.011	1.266	1.056	1.519
Average program duration per session (min)	≤29	Reference	–	–	–	–	–	–	–	–	–	–	–
	≥30	−1.745	0.765	0.022	0.175	0.039	0.782	−1.699	0.773	0.028	0.183	0.040	0.832
Fall efficacy		–	–	–	–	–	–	0.005	0.008	0.561	1.005	0.988	1.022
Outcome expectations for exercise		–	–	–	–	–	–	−0.029	0.021	0.155	0.971	0.932	1.011
Social support for physical activity		–	–	–	–	–	–	0.062	0.029	0.031	1.064	1.006	1.126
		*R*^2^ = 0.309, Hosmer and Lemeshow test x^2^ = 3.534, *p* = 0.897						*R*^2^ = 0.331, Hosmer and Lemeshow test x^2^ = 3.928, *p* = 0.864, Omnibus test of model coefficients x^2^ = 72.081, *p* < 0.001					

Model 1 included resident characteristics and facility factors. Model 2 additionally included psychosocial variables (fall efficacy, social support for physical activity, and outcome expectations for exercise). BMI, body mass index; ADL, activities of daily living.

### 3.2 Qualitative findings

The study included 12 participants, with 6 men and 6 women. The participants’ ages ranged from 81 to 94 years, and their length of stay in the facility varied from 3 to 21 months (Table 5). The reflexive thematic analysis of interview data yielded 274 codes, 13 subthemes, and 5 overarching themes.

**Table 5:** Characteristics of participants in 1:1 in-depth interviews.

Participant No.	Gender	Age (years)	Length of stay in the facility (months)	Current pain (Y/N)	ADL
P1	Man	81	7	N	8
P2	Man	84	6	N	8
P3	Man	84	14	N	9
P4	Man	90	13	N	8
P5	Woman	93	21	N	8
P6	Woman	92	12	Y	10
P7	Man	85	14	N	8
P8	Man	84	4	N	8
P9	Woman	87	13	N	9
P10	Woman	82	3	N	10
P11	Woman	94	12	Y	8
P12	Woman	80	8	N	9

ADL, activities of daily living.

#### 3.2.1 Theme 1: how past physical activity experiences shape current meanings of physical activity

For nursing home residents, perceptions and emotions surrounding physical activity were deeply rooted in how their earlier life experiences with physical movement continued to shape the meaning of activity in the present. Past experiences with sports, outdoor activities, and labor were reconstructed into feelings of loss, frustration, confinement, or, conversely, comfort and relief within the restricted environment of the facility and their declining physical abilities. Thus, their current attitudes toward physical activity were not solely a reflection of physical decline but emerged through the tension between their former physically active lives and their current limited circumstances.

##### 3.2.1.1 Sub-theme 1.1: memories of vitality and emotional distress in a constrained present

Some participants recalled that in their younger years, they routinely engaged in sports, hiking, and outdoor activities that brought a strong sense of vitality. They remembered physical activity as “a time when I affirmed my energy and identity.” However, after entering the facility, their opportunities for movement naturally decreased. Limited space, structured routines, and a management-centered daily schedule made it difficult to be active as they once had been. This discrepancy between past and present elicited emotions of sadness, frustration, isolation, and loss.

*“I used to love hiking, and even now my siblings take me out when they visit. I ran a business and drove everywhere—there wasn’t a place I did not go. I want to go out like that again, but being stuck here every day feels suffocating… Before, I could just go anywhere and see people. I never imagined I would end up living like this.”* (Participant 2).

##### 3.2.1.2 Sub-theme 1.2: memories of labor and the acceptance of rest

For some participants, physical activity was not associated with exercise but with a lifetime of physical labor. Those who had spent their lives working on farms, doing household labor, or engaging in strenuous occupations interpreted their admission to the facility as “finally a life of rest.” They felt no need to continue engaging in the kinds of physical activities they had performed throughout their lives. Rather than viewing activity as a means to maintain health, they perceived it as something unnecessary—an extension of labor they had already done enough of.

*“When I was young, I did not even know what exercise was—I just worked in the fields all day. (…omitted…) We harvested, loaded everything up, and by the time we got home it was dark. Working was my exercise back then. I do not want to do it anymore. I mean, look how old I am now.”* (Participant 12).

#### 3.2.2 Theme 2: physical activity as a means of restoring health and daily life

For nursing home residents, physical activity was understood not merely as exercise but as an experience that helped them restore their disrupted routines and weakened physical functions. Participants felt tangible changes—reduced pain, easier breathing, and less stiffness—when they moved their bodies, interpreting these moments as “feeling alive again” or “feeling my body come back.” Physical activity also elevated their emotional well-being through laughter, conversation, and social interaction, serving as a channel through which they could reconnect with others despite the isolation inherent in institutional life. Thus, physical activity emerged as a multifaceted restorative experience encompassing physical recovery, emotional release, and renewed social connection.

##### 3.2.2.1 Sub-theme 2.1: a sense of physical and emotional recovery

Participants experienced physical improvements through activity, such as reduced pain, smoother breathing, and decreased stiffness, which they described as their bodies “coming back to life.” As long-standing fatigue and discomfort eased, physical activity came to represent self-care and recovery, naturally reinforcing the belief that “you must move to stay alive.” Emotional benefits emerged as participants felt lighter, more relaxed, and more able to laugh and interact with others, making physical activity a crucial source of emotional vitality.

*“I actually used to wear diapers. I could not manage the bathroom at all myself. But lately I’ve been walking to the bathroom more often. In the beginning, the staff worried I’d fall, so they would bring the walker and stay with me. Then I walked two or three steps holding onto the bar by the bed. Now I can walk on my own. I feel that I’ve really improved.”* (Participant 1).

##### 3.2.2.2 Sub-theme 2.2: moments of affirming one’s value and agency

Beyond physical movement, physical activity provided participants with moments of restored self-efficacy and personal worth. Achievements such as “standing up on my own today” or “walking this far” became meaningful victories that helped rebuild trust in their bodies and reinforced their sense of identity.

*“After I exercise, I feel confident. I feel like I can do things because I’m healthy enough to move. If I just sat in my room doing nothing, I would feel weak and depressed. That’s why I exercise—to stay healthy and active.”* (Participant 4).

##### 3.2.2.3 Sub-theme 2.3: opportunities to reconnect with the world

Physical activity created natural encounters with other residents, staff, and outside instructors, fostering renewed social connection. As laughter, conversation, and interaction increased, physical activity served not only as exercise but as an opportunity to revive relational vitality. It functioned as a small “window to the world” that exceeded the repetitive and isolating nature of facility life.

*“There are people who come from outside to help us, and it makes me feel like my world gets a little bigger. Even when they listen to our not-so-good singing, it comforts us. It makes me realize we can still connect with the outside world.”* (Participant 2).

#### 3.2.3 Theme 3: motivating forces for continuing physical activity amid physical decline

Residents experienced pain, muscle weakness, and reduced balance due to aging, yet they held a sense of urgency that “things will get worse if I do not move.” Fear of further physical decline functioned not as a barrier but as a motivator to continue engaging in physical activity. In addition, encouragement and support from staff, family members, and volunteers provided an emotional foundation that helped them sustain their participation. In this way, the physical activity of nursing home residents was maintained through the interplay of two contrasting forces: fear of worsening physical function and supportive relationships in their environment.

##### 3.2.3.1 Sub-theme 3.1: continuing physical activity out of fear of further decline

Participants worried that their muscle weakness or gait instability would worsen if they remained inactive. This concern motivated them to keep moving, even when physical discomfort or fatigue made activity challenging. As several participants expressed, they felt the need to move “before my body stiffens even more” or “because if I stop, I will not be able to walk at all.”

*“When I see other patients in worse condition, I think, ‘I should not become like that.’ I wonder if I’ll be like that in ten years, and it weighs on my mind. But thankfully, this facility has its own programs, so participating helps pass the time. If you just lie down alone, you get more isolated. You have to manage yourself.”* (Participant 1).

##### 3.2.3.2 Sub-theme 3.2: sustaining physical activity through support from others

Support from staff, family members, and fellow residents played a crucial role in helping participants maintain activity. Encouragement, companionship, and being physically assisted fostered confidence and made it easier to continue exercising. For many, social support transformed difficult or intimidating activities into achievable ones.

*“When I go with others, they cheer, saying ‘Fighting!’ Even if someone cannot walk well, we all encourage each other. It gives me strength. And cheering them on is fun, too. Talking and laughing together helps a lot.”* (Participant 4).

#### 3.2.4 Theme 4: multilayered barriers that restrict physical activity

Institutionalized older adults encountered a wide range of constraints that limited their participation in physical activity. These barriers operated simultaneously at the physical, psychological, social, and environmental levels. Participants described not only personal challenges such as pain, physical decline, and fear of falling, but also external constraints including overprotective family members, relational pressure within communal living, monotonous programs, and the physical and institutional environment of the facility. Together, these multilayered factors shaped a context in which physical activity was difficult to initiate and maintain.

##### 3.2.4.1 Sub-theme 4.1: physical decline and fear of falling

Participants experienced pain, muscle weakness, and reduced balance that made initiating physical activity difficult. These physical limitations were closely intertwined with fear—fear of falling, being injured, or worsening their condition. Such concerns often led to psychological withdrawal and avoidance of physical activity.

*“My body does not allow it anymore. I get dizzy because of my age… I can walk, but not like a healthy person. That’s just how it is when you are older.”* (Participant 2).

##### 3.2.4.2 Sub-theme 4.2: withdrawal due to communal norms and relational pressure

Some participants felt self-conscious when they could not keep pace with others because of their functional limitations. Feeling that they might inconvenience others, they hesitated to join group activities. Statements such as “I do not go because I might cause trouble” reflected the relational pressure embedded in communal living. Differences in functional ability, activity speed, and concerns about burdening others all discouraged participation in group-based programs.

*“When others are doing things together, it’s hard for me. If I do not feel well, I want to lie down, but then it’s already time to go to the activity. The set schedules make me uncomfortable—I have to get up quickly, and that feels stressful.”* (Participant 7).

##### 3.2.4.3 Sub-theme 4.3: program limitations that do not reflect diverse needs

The physical activity programs offered in the facility were often repetitive and standardized, failing to consider the varying functional levels and preferences of residents. Participants frequently described these programs as “boring” or “too easy,” which lowered motivation and created internal barriers to participation.

*“They teach games and things like that. And exercise, but only for maybe 10 or 20 min. It’s so short. And the exercises are really easy—too easy to be fun.”* (Participant 11).

##### 3.2.4.4 Sub-theme 4.4: environmental and institutional constraints

Limited indoor space, restrictions on going outdoors, and the lack of exercise equipment reduced opportunities for physical activity. Institutional rules and safety-focused guidelines also restricted where, when, and how residents could move. These physical and policy-level constraints narrowed the possibilities for engaging in meaningful activity.

*“You have to exercise inside the boundary here. I cannot go out on my own without permission. I have to stay within this building. You cannot just decide what you want to do like you could outside. You have to follow the rules here and adjust to them.”* (Participant 4).

#### 3.2.5 Theme 5: expectations and needs for improved physical activity opportunities

Institutionalized older adults expressed a desire not merely for increased activity but for physical activity that aligned with their functional level, interests, and emotional needs. They hoped for programs that could help them break away from repetitive and monotonous routines and experience a sense of vitality and change. For these older adults, physical activity was expected to be not just a health-promoting behavior, but an experience that felt personally suitable, stimulating, and enjoyable.

##### 3.2.5.1 Sub-theme 5.1: tailored physical activity reflecting individual functional levels

Participants perceived the existing exercise programs as mismatched with their functional abilities, describing them as “too easy” or expressing a desire for “something a bit more challenging.” They wanted physical activity that incorporated both their abilities and preferences. The balance of challenge and achievement—activities that were not too difficult but still allowed a sense of progression—was particularly valued.

*“Walking is good, but everyone’s body is different. It would be better if they gave us something different depending on what we can do each day. If your legs are weak, then maybe move your arms instead. Doing the same thing all the time gets boring, so changing it to fit each person would be nicer.”* (Participant 3).

##### 3.2.5.2 Sub-theme 5.2: physical activity offering diverse environments and new stimuli

Participants wished for opportunities to experience even small moments of vitality and change by moving beyond monotonous indoor activities. Instead of programs confined to the same indoor space, they hoped for exposure to sunlight, fresh air, outdoor areas, new people, and new activities—environmental and emotional stimuli that could invigorate their daily lives.

*“We move around inside a lot. But going outside… we have not had that chance since I came here. It would be nice to walk outdoors. (Looking outside) When I see the fields, I think, ‘There must be grasshoppers, crickets out there.’ The hills nearby probably have katydids, too. Doing something outside that reminds me of those old days—that would be good.”* (Participant 1).

### 3.3 Integrated analysis of findings

**Figure 2:**
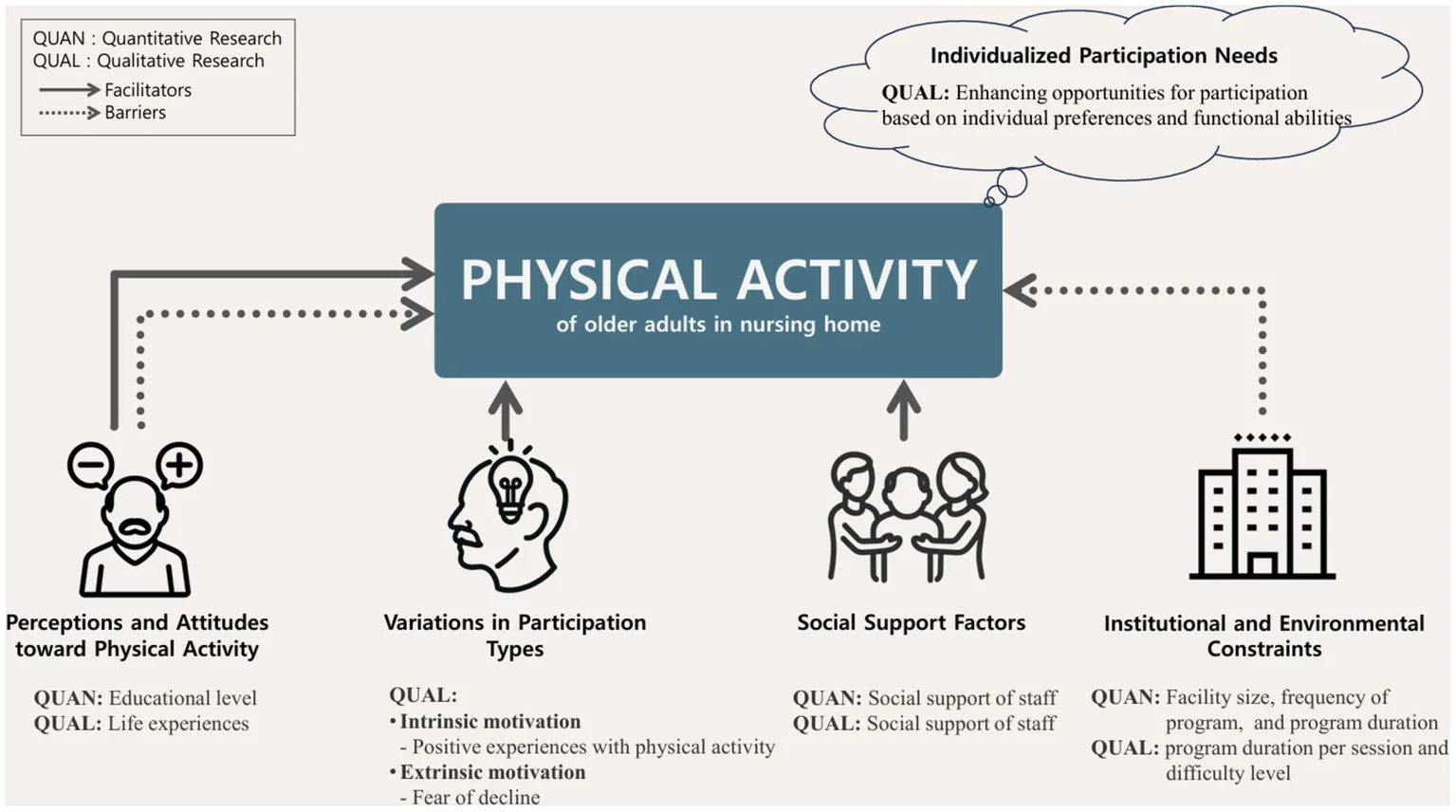
Joint display of quantitative and qualitative results on physical activity in nursing homes.

An integrated analysis of the quantitative and qualitative findings yielded five major integrated results (Figure 2).

First, regarding perceptions and attitudes toward physical activity, older adults’ life-course experiences emerged as key factors shaping how they perceived and engaged in physical activity. Quantitative analyses showed that personal characteristics previously reported as determinants of physical activity—such as gender, length of stay, and chronic conditions (6, 29)—were not significantly associated with physical activity frequency. Educational level was the only variable that demonstrated a significant association, with older adults who had completed middle school or higher reporting significantly higher levels of physical activity compared to those with no formal education.

To further elucidate these findings, qualitative data were integrated into the analysis. The integrated results indicated that residents’ perceptions and attitudes toward physical activity were shaped by their life-course experiences, which in turn played a critical role in influencing participation. Specifically, individuals who had been continuously exposed to informal and obligatory physical labor throughout their lives tended to perceive physical activity as routine work, leading to relatively negative attitudes and reduced participation. In contrast, residents with prior experiences in social and self-directed activities were more likely to hold positive perceptions of physical activity and to engage more actively. These life-course experiences reflected shared gendered patterns rooted in sociocultural contexts, underscoring the importance of considering residents’ biographical backgrounds and cultural histories when designing physical activity programs.

Second, the theme of diversity in participation motivation highlighted that multiple motivational pathways influenced engagement in physical activity. Quantitative analyses revealed that individual characteristics commonly identified as predictors of physical activity—such as functional decline, chronic illness, outcome expectations for exercise, and fall efficacy (6)—were not significantly associated with physical activity frequency, indicating limitations in explaining motivational dynamics through quantitative indicators alone.

Qualitative findings provided deeper insight into motivational processes, revealing that participation was driven not only by positive emotional experiences—such as expectations of health improvement, maintenance of self-esteem, and continuation of social relationships—but also by negative emotions, including fear of physical decline. These findings suggest that motivation for physical activity among nursing home residents is not shaped by a single factor but rather emerges from complex emotional experiences and life contexts. Accordingly, differentiated strategies—such as feedback that reinforces motivation, educational approaches that enhance the perceived meaning of physical activity, and activity designs that promote enjoyment and social interaction—are needed to address diverse motivational profiles.

Third, under the theme of social support factors, both physical and emotional support from facility staff emerged as influential in promoting residents’ physical activity. Quantitative analyses identified a significant association between social support and physical activity participation, though they were insufficient to explain the specific forms and mechanisms of support. Integrated qualitative analysis revealed that staff support extended beyond physical assistance to include encouragement and emotional reassurance that fostered participation. Residents also experienced support from family members and fellow residents, and these various forms of support functioned as critical contextual conditions enabling engagement in activities that would otherwise be difficult to perform independently. These findings underscore the need for staff training, feedback capacity building, and institutional support systems to strengthen social support mechanisms within facilities.

Fourth, facility and environmental factors were identified as having both constraining and facilitating effects on physical activity participation. Quantitative analyses showed that residents living in facilities with 30 or more beds reported lower levels of physical activity compared with those in smaller facilities (≤29 beds). In addition, a higher frequency of physical activity programs was associated with increased participation, whereas programs lasting longer than 30 min per session were significantly associated with reduced participation. These findings were generally consistent with prior studies indicating that larger facility size may lead to imbalances in activity opportunities (30) and that program frequency promotes participation (11). However, unlike previous studies in which programs were typically conducted for 30–45 min per session (11), the present study found that participation decreased when sessions exceeded 30 min, suggesting the need for contextual interpretation of this pattern. Qualitative findings suggested that when session durations exceeded residents’ functional tolerance, participants experienced physical burden and fatigue, which in turn discouraged continued engagement. This highlights the need to structure program duration in accordance with older adults’ endurance and attentional capacity.

Fifth, the theme of personalized participation needs reflected residents’ desire for greater opportunities aligned with their preferences and functional abilities. Because such needs were difficult to capture quantitatively, qualitative inquiry was undertaken. The findings indicated that residents lost interest or experienced frustration when activities did not match their preferences or abilities, whereas they demonstrated active engagement when activities were well aligned—particularly expressing strong interest in outdoor activities. These results support the necessity of choice-based, individualized program designs and participant-centered approaches (Table 6).

**Table 6:** Integrated findings on physical activity among older adults in nursing homes.

Higher-order themes	Associated factors	Quantitative findings	Qualitative findings	Integrated interpretation and implications
Perceptions and attitudes toward physical activity	Life experiences	No statistically significant difference between men and women. Compared with residents without formal education, those with middle school and high school or higher education were 3.194 times (95% CI: 1.228–8.310) and 3.936 times (95% CI: 1.543–10.042) more likely to engage in physical activity, respectively.	Held positive perceptions of physical activity, grounded in past experiences with social and leisure activities. Viewed physical activity negatively due to lifelong labor-centered experiences.	Approaches must consider gender roles and life-course experiences.
Diverse types of motivation for participation	Intrinsic motivation	No statistically significant findings in outcome expectations for exercise.	Positive emotional experiences—such as health improvement, enhanced self-esteem, and maintenance of relationships—encouraged continued participation.	Programs should incorporate components that provide positive feedback and emotional benefits.
	Extrinsic motivation	No corresponding quantitative findings.	Participation driven by anxiety about functional decline.	Educational approaches to increase awareness of the benefits of activity, combined with enjoyable and socially engaging components, are needed.
Social support factors	Physical and emotional support from staff	Higher social support increased physical activity participation (OR = 1.064, 95% CI: 1.006–1.126).	Staff encouragement and assistance played a central role in maintaining residents’ motivation to participate.	Strengthening staff education, feedback-based training, and institutional support is essential.
Facility and environmental constraints	Lack of individualization due to facility size	Residents in facilities with ≥30 beds had lower participation than those in facilities with ≤29 beds (OR = 0.159, 95% CI: 0.049–0.515).	Larger facilities tended to offer standardized programs with limited individualized feedback.	Facility-size–specific strategies and individualized support systems are needed.
	Program frequency, duration, and difficulty	Frequency of physical activity programs per week (OR = 1.266, 95% CI: 1.056–1.519) Program duration per session ≥30 min was associated with lower participation (OR = 0.183, 95% CI: 0.040–0.832).	More frequent program offerings increased opportunities for engagement and encouraged repeated participation. Longer or overly intensive sessions discouraged participation.	More frequent programs promoted engagement, and session duration should align with older adults’ endurance and attention span.
Individualized participation needs	Expanding opportunities aligned with preferences and functional level	No corresponding quantitative findings.	Residents expressed frustration or boredom when activities did not match their ability or preferences, and showed strong interest in personalized or outdoor programs.	Choice-based and individualized program design is needed to enhance engagement and resident-centered care.

## 4 Discussion

This study employed a mixed-methods approach to comprehensively examine nursing home residents’ perceptions of and needs related to physical activity. Although the regression model derived from quantitative analysis demonstrated statistical significance and acceptable model fit, it also suggested that factors beyond those included in the model may influence physical activity participation. Accordingly, qualitative inquiry was conducted to complement and contextualize the quantitative findings, enabling a more nuanced understanding of residents’ experiences. This discussion focuses on the integrated interpretation of quantitative and qualitative results and their key implications.

First, analysis of residents’ perceptions and attitudes toward physical activity revealed differences shaped by life-course experiences. No statistically significant differences in physical activity levels were observed by gender, contrasting with prior studies reporting gender-based differences (29, 31). Integrated qualitative findings indicated that residents’ perceptions and attitudes toward physical activity were formed through lifelong sociocultural environments and experiences, which influenced participation patterns.

These life-course experiences were shaped within sociocultural contexts that assigned distinct roles to men and women, resulting in relatively similar experiential patterns within each gender group. Men and women have historically accessed different social resources due to gender role socialization and structural opportunities (32), which may translate into differences in health behaviors—including physical activity—among both community-dwelling and institutionalized older adults (31). Prior studies have similarly reported that men residents with self-directed activity experiences tend to show greater interest and participation in physical activity, whereas women residents often perceive nursing homes as spaces of rest after caregiving responsibilities and engage more in socially oriented activities (29).

The absence of significant gender differences in quantitative findings may be attributable to the characteristics of physical activity programs in nursing homes. Many programs incorporate not only physical components but also cognitive and emotional elements (33), blurring the distinction between physical and social activities. As a result, gender-based preferences and participation tendencies may not be fully captured by quantitative measures such as activity frequency.

Similarly, education level was not significantly associated with physical activity frequency in the bivariate analysis; however, it emerged as a significant predictor in both hierarchical logistic regression models. This pattern may reflect a confounding control effect, whereby the relationship between education level and physical activity was partially masked by differences in residents’ health status, functional ability, and facility characteristics in the bivariate analysis, but became apparent after these factors were statistically controlled in the multivariable models. This independent effect may be partially explained by the tendency for individuals with higher education levels to possess greater health literacy and awareness of the benefits of physical activity, which may facilitate participation among nursing home residents (34).

Taken together, these findings underscore the need for integrated strategies that consider broader life-course experiences and individual characteristics when developing physical activity programs for nursing home residents (35). In particular, tailored approaches that address residents’ prior experiences and perceptions of physical activity, shaped by sociocultural and life-course contexts, are essential to promote sustained engagement. In this context, the tendency among residents with agrarian or industrial backgrounds to perceive physical activity as an unnecessary extension of labor has important practical implications. Healthcare administrators should reframe physical activity programs away from the concept of “exercise,” repositioning them as meaningful practices connected to residents’ life experiences (35). For residents with agrarian backgrounds, horticultural therapy may serve as an effective alternative, whereas ADL-based functional physical activity may be more appropriate for those with industrial or manufacturing backgrounds (36). Incorporating familiar Korean traditional games, such as Tuho and Yutnori, into physical activity programs can also naturally recontextualize movement as enjoyable leisure rather than labor. In particular, integrating gamification elements, such as points, rewards, and progress indicators, into physical activity programs has been shown to systematically alleviate negative perceptions of monotonous exercise and meaningfully enhance motivation, enjoyment, and engagement, representing a practical and scalable strategy for promoting physical activity among nursing home residents (37). Additionally, staff who identify individual residents’ occupational backgrounds and introduce physical activity using purpose-oriented language connected to residents’ past experiences may effectively facilitate greater participation (38).

Second, analysis of participation motivation revealed diverse motivational profiles among residents. Quantitative analyses showed no significant associations between physical activity and individual characteristics such as functional decline, chronic illness, outcome expectations, or fall efficacy (6). Qualitative findings indicated that intrinsic motivation was strengthened when residents experienced positive emotions through physical activity, including feelings of physical recovery, enhanced self-esteem, and sustained social relationships. These experiences reinforced perceptions of competence and efficacy, aligning with prior research demonstrating that satisfaction derived from activity enhances autonomy, competence, and relatedness, thereby promoting voluntary engagement (39).

Conversely, extrinsic motivation also played a role, driven by anxiety about aging, fear of functional decline, and perceptions of physical activity as part of daily routines within the facility. While some studies suggest that negative aging attitudes hinder participation (39), others posit that such perceptions may stimulate motivation for health maintenance (40). The findings of this study more strongly support the latter interpretation. These results highlight the need for differentiated, motivation-sensitive approaches that reflect residents’ emotional experiences and life contexts, as well as the dynamic nature of motivation (41).

Third, social support—particularly from facility staff—emerged as a critical facilitator of physical activity participation. Quantitative analyses identified staff-provided social support as a significant predictor of participation, while qualitative findings revealed that staff support functioned not only as physical assistance but also as emotional encouragement that enabled participation. Specifically, simple verbal encouragement such as “fighting!” or “you are doing well,” during walking routines provided tangible motivational boosts, and staff who actively joined residents during exercise, rather than merely directing them, made participation feel emotionally safe and comfortable. Proactive outreach, such as visiting residents’ rooms to encourage movement, served as an important trigger for participation, and specific praise for residents’ contributions enhanced their sense of competence and self-efficacy. These findings align with prior research characterizing staff as facilitators of behavioral change and enablers of participation among residents with functional limitations (42). Moreover, the results underscore the importance of organizational and institutional environments that support staff in integrating physical activity into routine care practices through training and systemic support (43).

Fourth, structural characteristics of facilities and programs functioned as both contextual constraints and facilitators of physical activity participation. A larger facility size was associated with reduced participation, which can be interpreted through two complementary mechanisms. First, smaller facilities may have relatively higher staff-to-resident ratios, facilitating individualized support and emotional encouragement for residents. Second, the small-scale, home-like environment created by smaller facilities may inherently foster spontaneous and autonomous movements among residents. Prior research indicates that small-scale, accessible spaces promote spontaneous activity engagement even without staff guidance (44), and that maintaining autonomy in activity choice and space use has been reported to positively influence older adults’ psychological well-being and sustained engagement (45). Notably, facility size remained a significant independent predictor even after controlling for staff support and other variables in the regression model, suggesting that the home-like environment of smaller facilities, beyond staff-to-resident ratios alone, may serve as a more fundamental facilitator of spontaneous and autonomous participation. However, as this study employed a cross-sectional survey design, definitively determining the relative contribution of each mechanism is beyond the scope of the present study. In contrast, a higher frequency of physical activity programs was associated with increased participation, indicating that repeated exposure and expanded opportunities may promote engagement. However, longer program duration per session was linked to decreased participation, underscoring the importance of aligning session length with residents’ physical and cognitive capacities. While the qualitative findings attributed this pattern primarily to physical burden and fatigue, attentional and cognitive fatigue warrant consideration as additional contributing factors. Physical activity programs in nursing home settings often rely on standardized and repetitive formats with limited variation in content (46), and in such contexts, cognitive fatigue may progressively accumulate as sessions are prolonged, leading to attentional decline and disengagement even in the absence of substantial physical exertion. This is supported by evidence that repetitive and unstimulating tasks can progressively impair sustained attention and induce cognitive fatigue (47). However, cognitive fatigue is more likely to emerge when extended session lengths are combined with monotonous, repetitive program content rather than being an inherent consequence of session duration. Given that this study employed a cross-sectional survey design, the relative contribution of each mechanism cannot be directly determined, and this limitation has been explicitly acknowledged. Taken together, both facility size and session duration point to a common underlying issue: the extent to which the environment and programs are designed to align with residents’ individual capacities and preferences. Just as small-scale, home-like environments foster autonomous participation, programs that are individualized to residents’ cognitive and physical capacities are more likely to sustain long-term engagement. This suggests that both environmental context and program individualization should be considered simultaneously in the design of physical activity programs for nursing home residents (48, 49).

Finally, physical activity participation was more meaningful and sustainable when aligned with residents’ preferences and functional capacities. Rather than being treated as a standardized service, physical activity should be reconceptualized as a personalized experience reflecting individuals’ life contexts and choices. Prior research supports the benefits of individualized physical activity approaches in improving physical and emotional health outcomes (42, 50). However, practical strategies for implementing individualized programming remain limited in nursing home settings (11), highlighting the need for actionable operational frameworks that enable personalized participation within constrained environments.

### 4.1 Limitations

Nursing home residents often have limited capacity for physical activity due to cognitive impairment, stroke, fractures, and severe chronic conditions (5). Considering these characteristics, the fact that this study included only residents who were able to engage in physical activity presents a limitation to the generalizability of the findings. In particular, given that the vast majority of the institutionalized older population suffers from cognitive decline or severe frailty (51), the findings of the present study may not be directly generalizable to more vulnerable nursing home residents with severe cognitive impairment or physical frailty. Future research should therefore include residents with diverse cognitive and functional levels to explore perceptions and attitudes toward physical activity from a more comprehensive perspective.

Furthermore, the quantitative component of this study examined physical activity based only on the number of times residents participated in physical activity over the past week, which may be subject to recall bias and may not fully capture the multidimensional nature of physical activity in older adults. Physical activity is a multidimensional construct encompassing frequency, duration, intensity, and type, all of which may influence its meaning and health effects (52). Therefore, future studies should incorporate objective measures, such as accelerometry, to more accurately track physical activity patterns and minimize the risk of measurement bias while adopting approaches that incorporate these multiple dimensions of physical activity.

Finally, in this study, although nursing homes were selected by considering the size during recruitment, there are limitations to generalizing the results, as the participating nursing homes may not fully represent all nursing homes.

Despite these limitations, this mixed-methods study reaffirmed the importance of staff social support in promoting physical activity among nursing home residents (42), but also identified aspects that have been underexplored in prior research. These include differences in perceptions and attitudes shaped by life history, diverse motivational factors, the specific influence of environmental constraints such as program structure, and expectations regarding physical activity. Through these findings, the study offers deeper insights into physical activity among nursing home residents and provides foundational knowledge for developing and implementing programs that can effectively enhance their participation in physical activity.

## 5 Conclusion

This study examined nursing home residents’ perceptions and needs regarding physical activity, with the aim of informing future approaches to physical activity interventions in long-term care settings. The findings revealed that residents’ perceptions and attitudes toward physical activity differed by lifetime experiences, and that they engaged in physical activity based on diverse motivational factors. Support from staff and the structural characteristics of activity programs were identified as key influences on participation. Additionally, residents expressed clear expectations for individualized programs tailored to their functional levels and personal preferences.

To effectively promote physical activity among nursing home residents, it is essential to design personalized interventions that reflect differences in perceptions by life history, motivational profiles, functional abilities, and individual preferences. Such program designs should not only present activities but also incorporate structures that sustain residents’ interest and ensure autonomy in choosing activities. Furthermore, because staff play a central role in facilitating physical activity participation, systematic training and ongoing support for staff are crucial for successful implementation. Finally, to ensure that individualized activities can be provided consistently and that participation opportunities can be expanded, practical and operationally feasible systems must be established within long-term care facilities.

## Data Availability

The raw data supporting the conclusions of this article will be made available by the authors, without undue reservation.
